# Functional connectivity dynamics slow with descent from wakefulness to sleep

**DOI:** 10.1371/journal.pone.0224669

**Published:** 2019-12-02

**Authors:** Mazen El-Baba, Daniel J. Lewis, Zhuo Fang, Adrian M. Owen, Stuart M. Fogel, J. Bruce Morton

**Affiliations:** 1 Faculty of Medicine, University of Toronto, Toronto, Ontario; 2 Department of Psychology, Western University, London, Ontario; 3 Brain and Mind Institute, Western University, London, Ontario; 4 School of Psychology, University of Ottawa, Ottawa, Ontario; 5 The Royal’s Institute for Mental Health Research, University of Ottawa, Ottawa, Ontario; 6 Brain & Mind Institute, University of Ottawa, Ottawa, Ontario; University of British Columbia, CANADA

## Abstract

The transition from wakefulness to sleep is accompanied by widespread changes in brain functioning. Here we investigate the implications of this transition for interregional functional connectivity and their dynamic changes over time. Simultaneous EEG-fMRI was used to measure brain functional activity of 21 healthy participants as they transitioned from wakefulness into sleep. fMRI volumes were independent component analysis (ICA)-decomposed, yielding 42 neurophysiological sources. Static functional connectivity (FC) was estimated from independent component time courses. A sliding window method and *k*-means clustering (k = 7, L2-norm) were used to estimate dynamic FC. Static FC in Wake and Stage-2 Sleep (NREM2) were largely similar. By contrast, FC dynamics across wake and sleep differed, with transitions between FC states occurring more frequently during wakefulness than during NREM2. Evidence of slower FC dynamics during sleep is discussed in relation to sleep-related reductions in effective connectivity and synaptic strength.

## Introduction

The transition from wakefulness to sleep is highly organized into a continuum of heterogeneous stages that change dynamically over the course of a single night and is associated with marked alterations in brain function and cognition. There is, for example, a marked reduction in long-range inter-cortical functional connectivity (FC) in the descent from alert wakefulness to deep, slow-wave sleep [1–3], and a dampening of inter-regional effective connectivity dynamics [4]. This reduced connectivity may represent processes which support the known functions of sleep, including reduced sensory processing [5–6], the disengagement of executive control [7], synaptic homeostasis [8], and sleep-dependent memory processes [9–12].

One important means of studying the brain’s FC and its relation to sleep is through simultaneous electroencephalography (EEG) and functional magnetic resonance imaging (fMRI). The method is particularly powerful as EEG provides a gold-standard means of identifying qualitatively distinct sleep stages and fMRI provides a whole-brain, albeit indirect, measure of brain activity in the form of the blood oxygenation level dependant (or BOLD) signal. To date, most studies of functional connectivity (FC) and sleep have compared inter-regional BOLD correlations (i.e., static FC) observed during wakefulness with those observed during sleep [13–15], an analysis strategy that, by design, compares time-invariant properties of FC across wake and sleep. However, FC, like sleep itself, is highly dynamic and subject to considerable variation over relatively short time scales [16–17]. Conventional approaches therefore potentially overlook differences in inter-regional connectivity dynamics that would be expected given known changes in synaptic strength and effective connectivity that occur during the transition from wakefulness to sleep. Thus, the current study examined changes in FC dynamics during normal wake to sleep transitions through the use of simultaneous EEG-fMRI.

Neurologically intact healthy adult individuals were scanned at 3-Tesla as they transitioned from wakefulness to sleep. Sleep status was monitored throughout the scanning period via simultaneously collected EEG. Our analysis focused on time-dependent, or dynamic, changes in inter-regional FC measured by fMRI, rather than time-invariant FC. Whole-brain patterns of FC alternate between periods of stability and instability, potentially reflecting transitions into and out of successive, qualitatively distinct, connectivity states [16]. Such time-dependent, or dynamic changes in whole-brain FC can be readily observed via fMRI and quantified by means of sliding-window methods [18]. Resulting metrics, including the number of transitions and the inter-transition interval, reflect how frequently connectivity states change over time, and are associated with variation in participant arousal and attentiveness [19–20]. As such, use of dynamic measures of inter-regional coupling to compare sleep and wake functional imaging data was well motivated given the objectives of the current study.

In this study, we investigated temporal characteristics of functional connectivity during wakefulness and sleep. Using a sliding-window analysis of functional connectivity dynamics, we hypothesized that whole-brain functional connectivity state transitions would occur less frequently during sleep than during wakefulness. These insights would be overlooked by conventional methods (i.e., static FC) that assume a stable functional connectivity configuration over time and may potentially serve as biomarkers for altered states of awareness such as anaesthesia, persistent vegetative state, and coma.

## Methods

### Participants

Thirty-five healthy right-handed adults [20 female, ages 20 to 34 years (M = 25.6, SD = 3.6)] participated in this study. All participants were screened based on a-priori exclusion criteria including, shift workers, history of head injury or seizures, diagnosis of a sleep disorder, neurological, psychiatric or chronic physical pain, high body mass index (> 25), taking prescription medications, and excessive caffeine, nicotine, or alcohol consumption (see S1 File). Participants were asked to comply with a strict sleep schedule for seven days prior to scanning that required them to maintain regular sleep-wake cycles (i.e., sleep-time between 22h00 and 24h00, and wake-time between 07h00 and 09h00), and to refrain from taking day-time naps. Participants were instructed to complete a sleep diary detailing their consumption of stimulants (e.g., caffeine) and their sleep/wake times, and to record any sleep complaints. Wrist actigraphy (Actiwatch 2, Philips Respironics, Andover, MA, USA), worn on the non-dominant wrist, was used to assess compliance to the sleep schedule. All participants were screened at least one week before scanning. Imaging procedures occurred between 22h00 and 24h00. The Western University Health Science Research Ethics Board provided ethics approval for this study. Participants provided written consent before taking part in the study.

### Connectivity matrices and analyses

**Fig 1:**
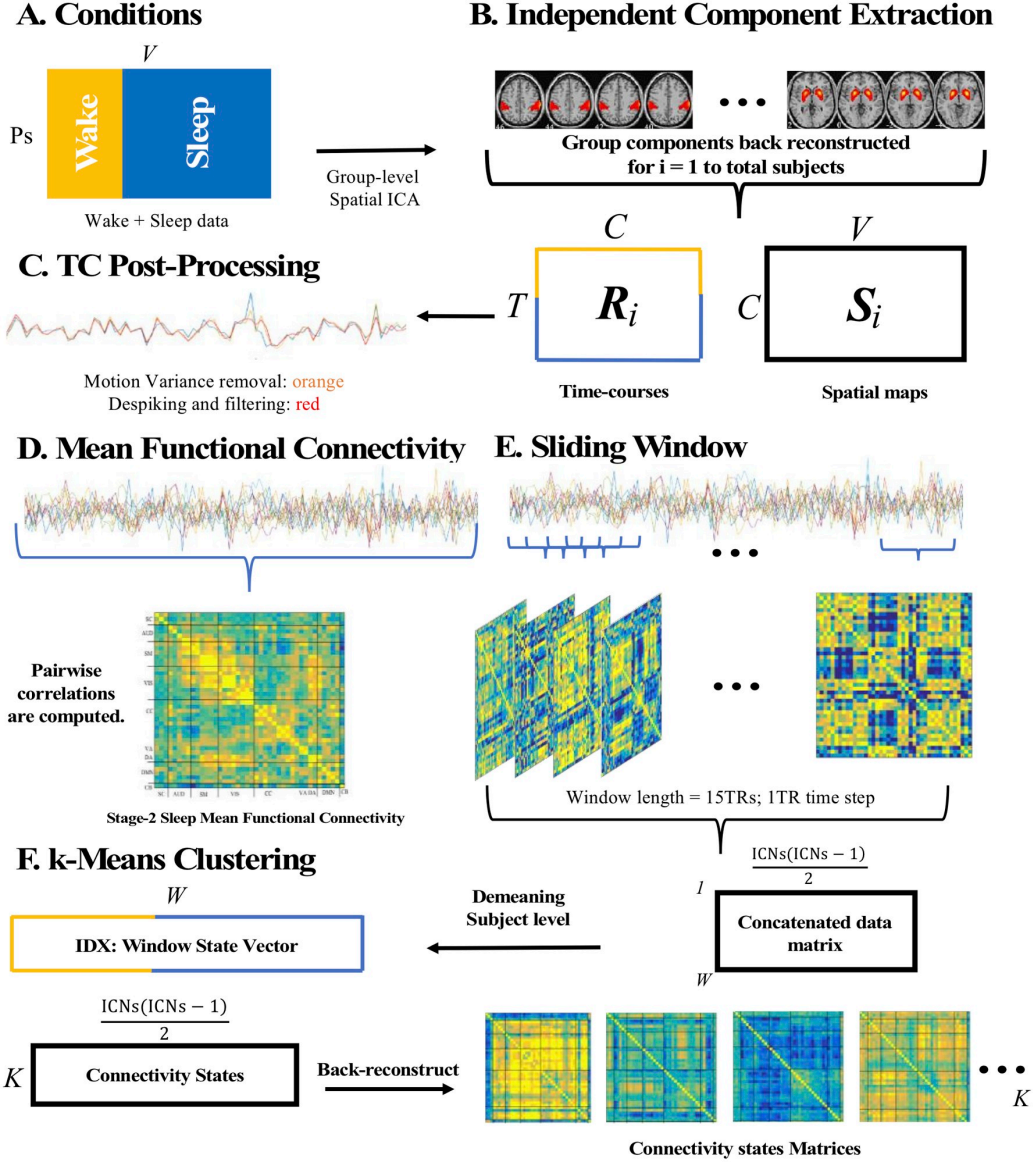
Step-by-step analysis illustrated. *A*, Subjects wake and sleep volumes where organized and submitted to group-level spatial independent component analysis (ICA). *B*, ICA was performed using the GIFT toolbox implemented in MATLAB; 42 neurophysiologically plausible sources were selected and sorted into functional families. GICA 1 back-reconstruction was used to estimate the time courses (Ri) and spatial maps (Si) for each subject. *C*, All time courses (TCs) were post processed by removing subject motion variance, despiking, and filtering. *D*, TCs were used to estimate mean FC in Wake and NREM2 by computing pairwise correlations between all ICs. *E*, Dynamic FC was estimated using a sliding window approach (window width = 15 TRs, time step = 1 TR) resulting in windowed correlation matrices. Correlation matrices were vectorized and concatenated into a large data matrix of all IC-to-IC pairwise correlation values over time. The concatenated data matrix was submitted to k-means clustering (*F)*. k-means clustering was performed to extract recurrent features of the data. A k-7 solution was selected which resulted in a matrix with rows equivalent to the number of connectivity states (7) and columns equivalent to the number of unique IC-IC correlations (861). Each row representing a connectivity states was back-reconstructed into a matrix format to visualize coupling relationships between ICs. An IDX vector, a window state label vector, unique for each subject in Wake and NREM2 was computed revealing window classification under one of the 7 connectivity states.

Details pertaining to EEG-fMRI acquisition and data preprocessing can be found in S1 File. Further, an overview of the analysis is depicted in Fig 1. Of the 35 participants, 21 were included in the analysis who had sufficient sleep (i.e., minimum of 5 minutes of consolidated sleep per sleep stage), that were not contaminated by motion artifacts (translation cut-off = 1.5mm, rotation cut-off = 1.5 degrees). Only five participants had a minimum of 5 minutes of consolidated sleep in NREM3 sleep; therefore, the analysis focuses on only wakefulness and NREM2.

**Fig 2:**
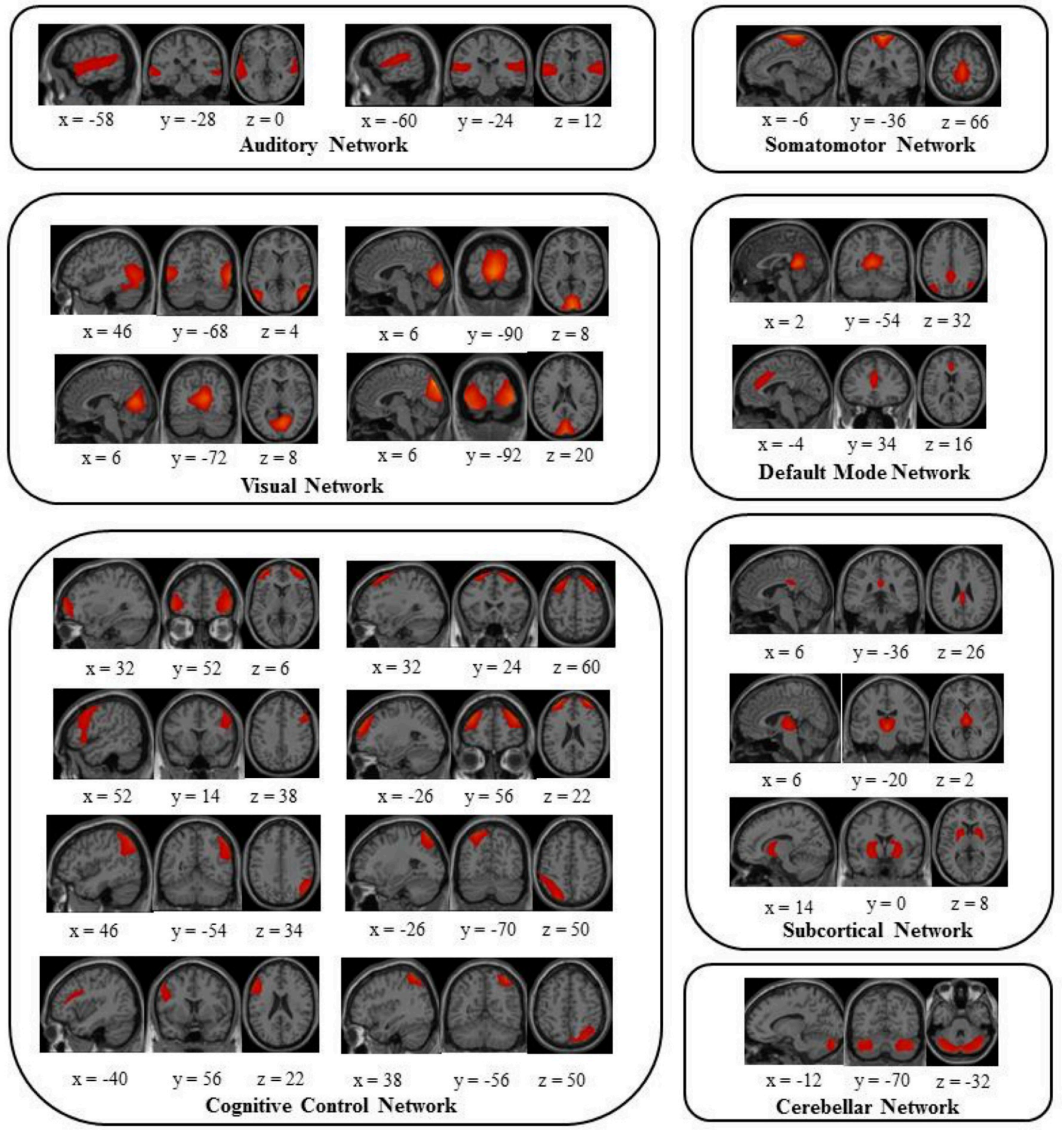
42 neurophysiologically plausible independent components (ICs). 42 neurophysiologically plausible independent components (ICs). Were divided into groups and arranged based on their spatial and functional properties. A total of seven functional families were identified including the auditory, somatomotor, visual, default mode, cognitive control (including the dorsal and ventral attention networks), subcortical, and cerebellar networks. ICs are displayed on sagittal, coronal, and horizontal slices on a cortical surface implemented in MANGO.

All wake and sleep volumes were submitted to a group-level spatial ICA implemented in the GIFT toolbox (http://mialab.mrn.org/software/gift/) in MATLAB to decompose data into functional networks. In the first step, Principal Components Analysis (PCA) reduced subject-specific data to 65 components. Data were then aggregated across participants. The Infomax algorithm was then applied to obtain 65 maximally independent components (ICs). A high model order was used to optimally separate noise and source components, as well, to ensure a spatially fine-grained parcellation of cortical and subcortical brain regions [21]. Subject ICs were then back reconstructed. Of the 65 ICs, 42 were identified as neurophysiological plausible sources (see Fig 1) by two observers based on an independent visual inspection of the spatial maps, using previously published criteria [18]. The 42 ICs were classified under seven functional networks that include the subcortical (SC), auditory (AUD), somatomotor (SM), visual (VIS), cognitive control (CC) [including dorsal attention (DA) and ventral attention (VA)], default mode (DMN), and cerebellar (CB) networks (see Fig 2).

IC time courses (TCs) were then further post-processed to maximize signal-to-noise ratio. First, estimates of translational and rotational motion and their temporal derivatives were regressed out of individual TCs using linear regression. Second, residualized TCs were despiked by replacing any spike greater than 3 standard deviations (STDV) with values equal to 3 STDV and low-pass filtered using a Butterworth filter with a cut-off frequency of 0.15 Hz. Last, a Fisher z-transformation was applied, and the resultant TCs were used for further analyses.

Building on a previously published approach, dynamic FC was estimated with a sliding window approach [18]. To increase sensitivity to condition-specific connectivity dynamics (for discussion, see [22]), individual windowed FC matrices were demeaned by removing subject-specific mean FC from each FC window. All demeaned windowed FC matrices were then transformed into vectors and concatenated, such that rows of the resulting matrix represented FC windows and columns represented unique IC to IC couplings. This matrix was then clustered using a k-means algorithm and a squared Euclidean distance metric (S1 File). Rows of the resulting C-matrix represented cluster centroids and were interpreted as recurring group-level FC patterns. Elements of the resulting IDX (or state label) vector reflected the classification of each windowed FC matrix into one of *k* states. Subject-specific IDX vectors were parsed out of the group-level IDX vector, and used to estimate four different dynamic connectivity metrics including the number of transitions, inter-transition interval, frequency of connectivity state expression, and mean dwell time (see SI for more information).

## Results

### Static FC

**Fig 3:**
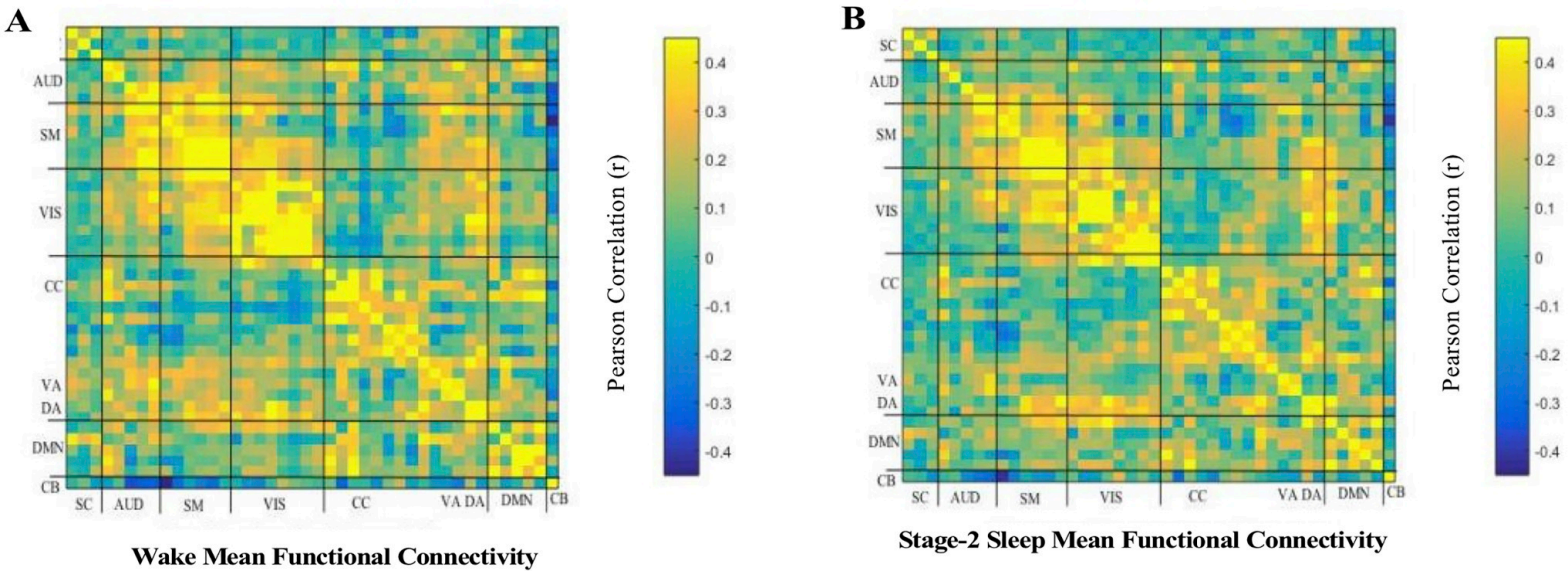
Mean functional connectivity in Wake and NREM2. Mean functional connectivity in Wake (A), and NREM2 (B). Within defined boundaries around the diagonal, positive correlations indicate strong coupling relationships between ICs that were classified within the same functional family. Off-diagonally, weaker connectivity can be found between functional families.

**Fig 4:**
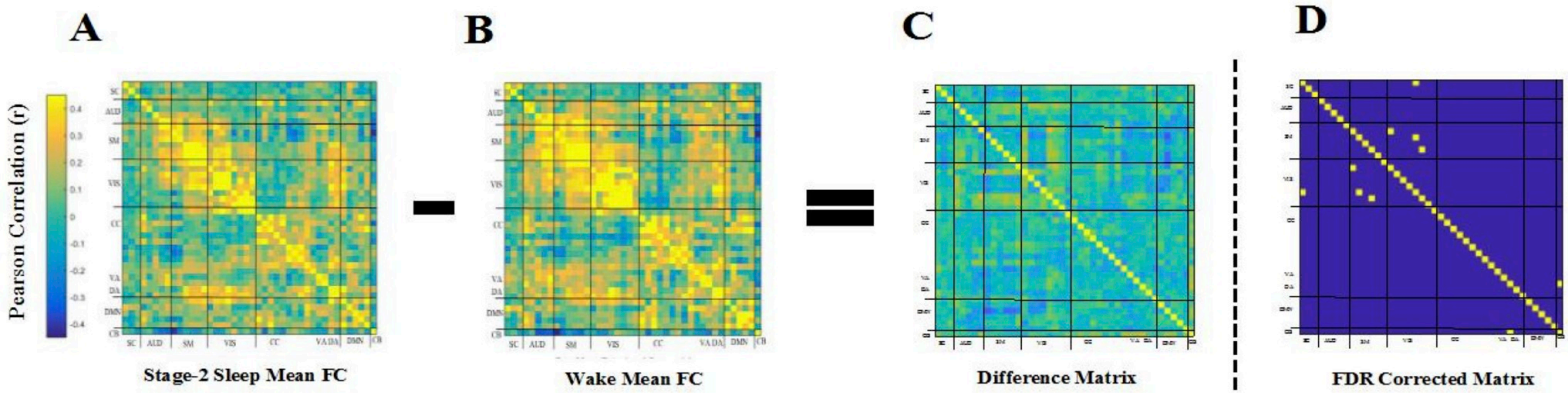
Difference matrix between wake and NREM2 FC matrices. Stationary Mean FC in Wake (B) subtracted from stationary mean FC in NREM2 (A) to produce a Difference Matrix (C). *t* tests were performed with the null hypothesis of zero correlation on the Difference Matrix (D). To correct for multiple comparisons, the false discovery rate (FDR) method was used with a *P* value of .01. t-tests confirmed that mean FC in Wake and NREM2 were similar with five correlation differences that were greater than 0.

Wake and sleep fMRI volumes from twenty-one participants (Mean age = 23.95, SD = 4.18, 13 Females) who had sufficient consolidated sleep (bare minimum of 5 minutes) were included in the analysis (see S1 File). Group-level spatial Independent Component Analysis (ICA) implemented in the GIFT toolbox (http://mialab.mrn.org/software/gift/) was conducted to decompose the data into functional networks. Subject-level time courses (TCs) were generated via back-reconstruction, and then filtered, detrended, and despiked. 42 neurophysiologically plausible sources were identified (Fig 2). Static FC in Wake and NREM2 were estimated by computing pairwise correlations between TCs of the 42 ICs from both conditions (see Fig 1 for an illustration). Resulting FC matrices were highly similar (spatial correlation; *r* = .865, *p* < .001; Fig 3), and marked by positive intra-network correlations and weaker inter-network correlations. To test for differences, we subtracted each participant’s Wake matrix from their NREM2 s matrix and tested resulting differences against 0 at the group level by means of *t* tests, FDR-corrected at *p* = .05 (Fig 4). Five couplings showed differences in FC across wake and sleep. These included a decrease in FC between during sleep in selected visual and subcortical ICs, selected visual and visual ICs, and selected cerebellar and ventral attention ICs.

### Dynamic FC

Our analysis of functional connectivity dynamics was modelled closely on the procedures of Allen et al. [18]. Dynamic FC was estimated using a sliding window method (see Fig 1 for an illustration) in which windowed correlation matrices were computed from TC segments, separately for wake and sleep data, demeaned by removal of subject-specific mean FC (see [22]) and concatenated (see Fig 1E). Parameterization of the sliding window method in the current analysis, including choice of window width (15 TRs or approximately 35s) and time step (1TR), followed precedents established by Allen et al. [18] and Hutchison and Morton [20]. *K*-means clustering was then applied to the concatenated set of windowed FC matrices to produce an unsupervised, or data-driven, identification of repeating functional connectivity states. For this, we followed the precedent of Allen et al. [18] and used a 7-category solution (i.e., *k* = 7). Owing to the fact subject-specific mean FC was removed from individual windowed FC matrices (following [22]), cluster centroids produced by the current analysis were markedly different than those previously reported by Allen et al. (2014). Results were largely unchanged across solutions based on different values of *k* (see S1 File).

### Dynamic metrics

Four measures of functional connectivity dynamics were computed separately for participants and conditions (i.e., Wake and NREM2). Two measures were repertoire-wide in that they were based on the complete set (or repertoire) of 7 states, and two measures were state-specific in that they were based on each of the 7 states separately. Repertoire-wide measures included: (a) the number of transitions, computed as the total number of state transitions evidenced by a participant in a condition corrected for the total number of windows in that condition for that participant; and (b) inter-transition interval, computed as the average number of consecutive instances of the same state before a transition to a different state. State-specific measures included: (c) frequency, computed as the proportion of one participant’s windows classified as instances of a particular state; and (d) mean dwell time, measured as the average number of consecutive windows classified as instances of a particular state.

**Fig 5:**
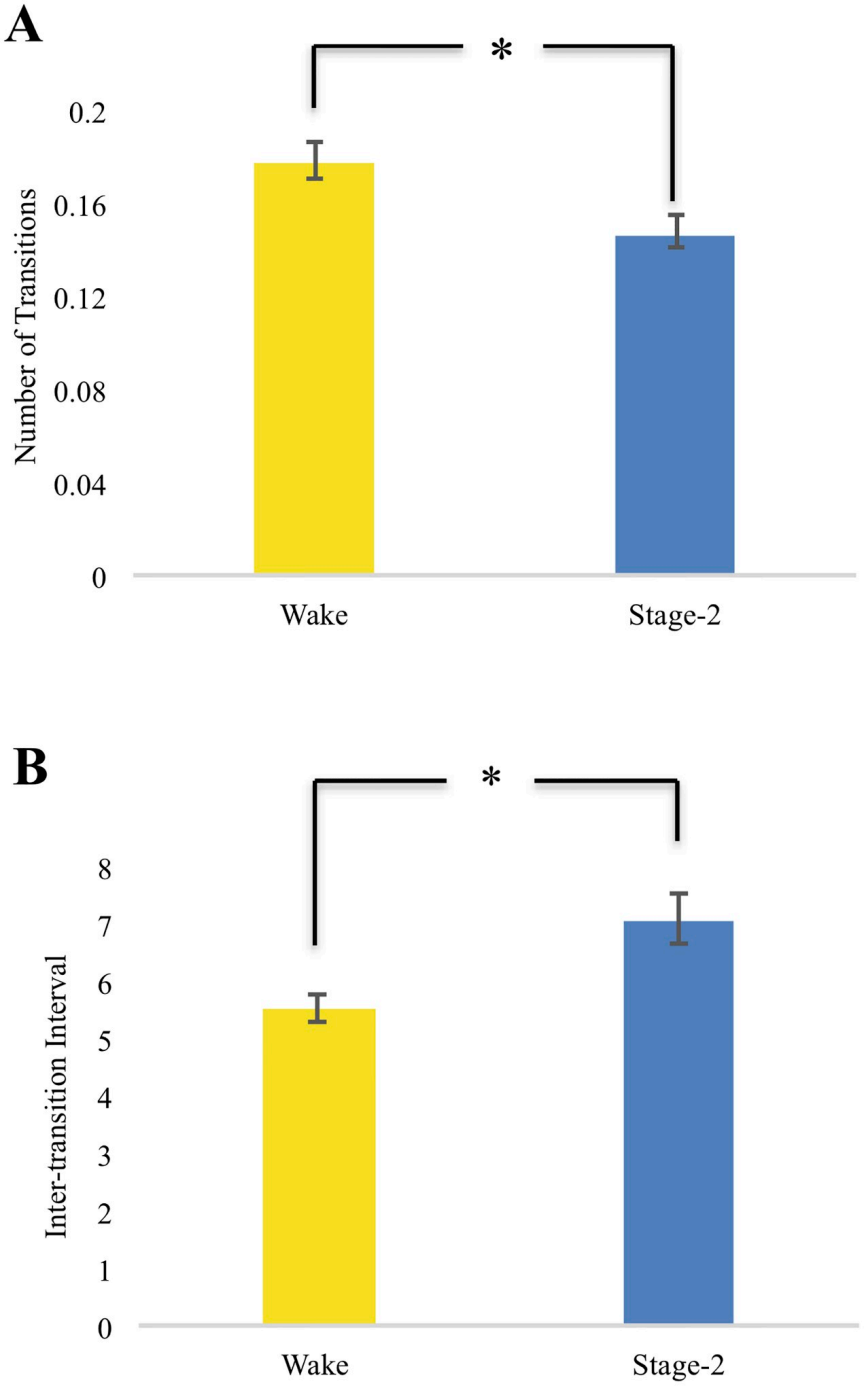
Mean number of transitions (NT) and inter-transition interval (ITI). A, Mean number of transitions (NT) in Wake and NREM2. Participants expressed more transitions in Wake (*M* = .18; *SD* = .036) than in NREM2 (*M* = .15; *SD* = .033) (*p* < .05). B, The inter-transition interval was significantly higher in NREM2 (*M* = 7.08; *SD* = 1.96) than in Wake (*M* = 5.52; *SD* = 1.10) (*p* < .01).

With respect to repertoire-wide measures, the number of transitions was lower during NREM2 (*M* = .15; *SD* = .033) than during wakefulness (*M* = .18; *SD* = .036; *t*(20) = -2.49, *p* < .05; see Fig 5A), and the inter-transition interval was longer during NREM2 (*M* = 7.08; *SD* = 1.96) than during wakefulness (*M* = 5.52; *SD* = 1.10; *t*(20) = -2.95, *p* < .01; see Fig 5B).

**Fig 6:**
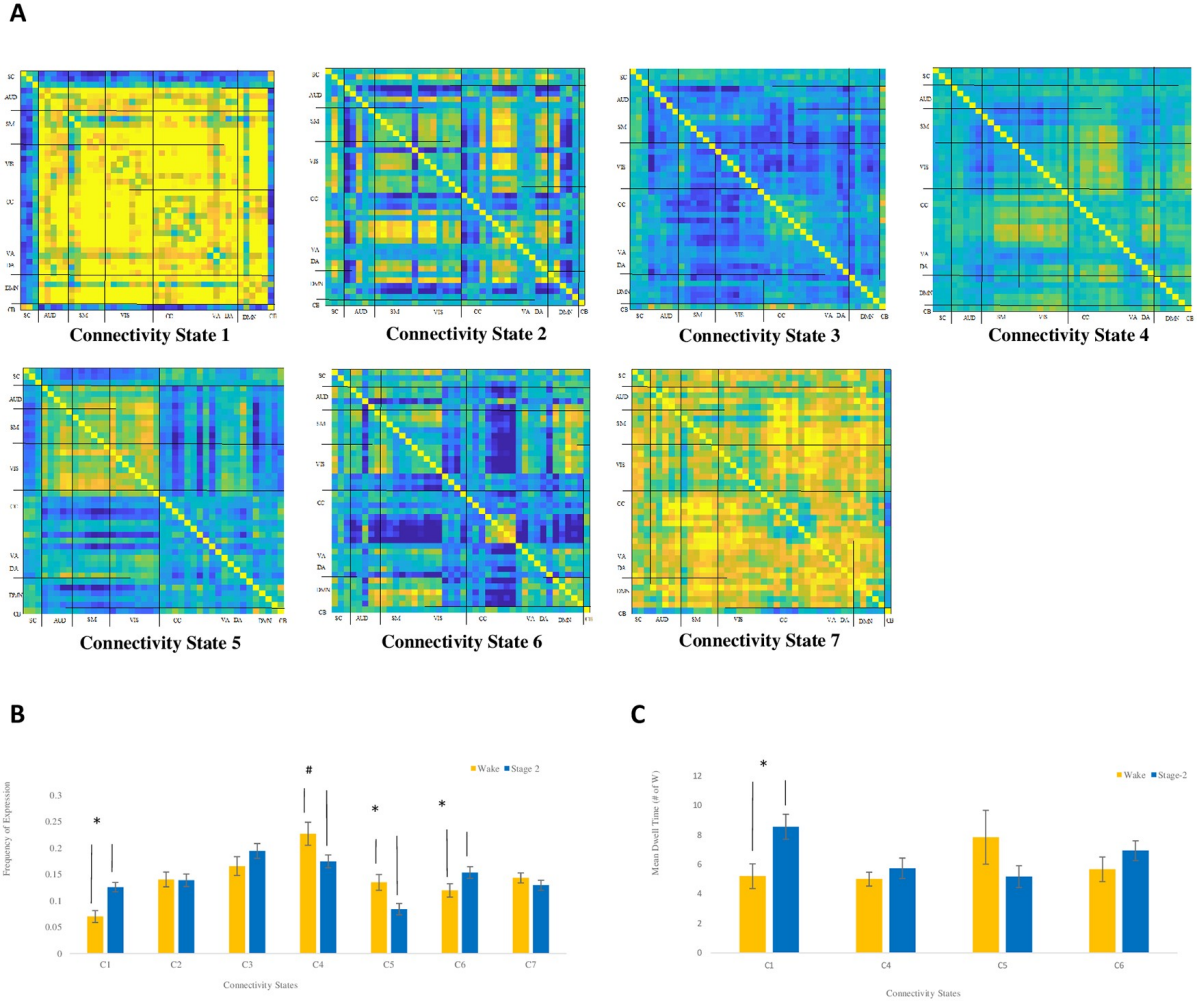
Cluster analysis, mean frequency, and mean dwell time. Clustering analysis revealed 7 connectivity states in Wake and NREM2 (A). B, mean frequency of state expression in Wake (yellow) and NREM2 (blue). The frequency of connectivity state-1 and 6 were significantly greater in NREM2 (*, *p* < .05). The frequency of connectivity state-5 was significantly greater in Wake (*, *p* < .05); the frequency of connectivity state-4 expression in wake was marginally significant when compared to NREM2 (#, *p* = .053). C, Mean dwell time for connectivity state-1 was significantly greater in NREM2 than in Wake (*, *p* < .05).

With respect to state-specific measures (i.e., frequency and mean dwell time), three of seven states showed a difference in frequency across Sleep and Wake (Fig 6B). For two states, frequency was higher during NREM2 than during Wake. These included State-1, a state marked by heightened cortex-wide connectivity and reduced connectivity between cortical and subcortical networks, and State-6, a state marked by reduced connectivity between cognitive control and default mode ICs. For a third state, State-5, frequency was higher during Wake than NREM2. This state was marked by heightened connectivity between auditory, visual, and somatomotor networks. The four remaining states did not show a difference in frequency across NREM2 and Wake. With respect to mean dwell time, only one state showed a difference across NREM2 and Wake, with State-1 exhibiting longer mean dwell time during NREM2 than during Wake (see Fig 6C).

## Discussion

Consistent with our predictions, qualitative changes in whole-brain functional connectivity occurred less frequently over time when participants were asleep compared to when they were awake. This sleep-related slowing of connectivity dynamics was most clearly reflected by a decrease in the number of transitions between qualitatively distinct connectivity states during sleep relative to wakefulness, and accordingly, by an increase in the length of time between transitions. These findings were robust across multiple clustering solutions (see SI for figures) and were not easily attributed to differences in non-neurophysiological artifacts such as motion and cardiac pulse rate (see SI for figures).

Repertoire-wide changes in connectivity dynamics, as reflected in the number of state transitions and the length of the inter-transition interval across sleep and wakefulness, were driven largely by changes in the dynamics of three individual states. Of particular interest were sleep-related increases in the frequency and dwell time of connectivity State-1, a state marked by increased inter-cortical connectivity and decreased cortical-subcortical connectivity relative to the participant’s own mean FC. This is consistent with differences in the mode of operation of cortico-subcortical circuitry in NREM2 compared to wake, whereby at the cellular level, the brain oscillates between up-states (involved in the generation of slow oscillations), separated by down-states (periods of synaptic quiescence) [23–26]. At the macroscopic level, this is reflected in what is termed the cyclic alternating pattern (CAP) [27]. In young adults, the CAP is in the time-scale that could be reflected in fMRI time-courses (e.g., ~4.5 sec) [28]. The CAP corresponds to different levels of arousal [29]. However, the link between CAP and the BOLD signal remains to be directly investigated. Despite this, we would speculate that the repertoire-wide changes in connectivity dynamics might reflect fluctuations in arousal, alternating between states, which are either characterized by inter-cortical connectivity or cortico-subcortical connectivity. This suggests that dynamic FC approaches may be useful to investigate the relationship between CAP and the BOLD signal, and its functional significance.

In terms of state-specific expression of connectivity states, evidence that dynamic transitions between qualitatively distinct connectivity states occur more frequently during wakefulness than sleep is broadly consistent with the view that dynamic exploration of connectivity state space provides a foundation for cognitive function [30], and parallels earlier reports linking variation in transition rate and dwell time to variation in cognitive capacity related to arousal, age, and neurophysiological integrity [20, 31]. The findings are also broadly consistent with the idea that wakefulness is a period of global synaptic potentiation, whereas NREM sleep supports synaptic homeostasis [8]. Synapses are subject to widespread changes in composition and signalling during the transition from wakefulness to sleep, including the down-regulation and dephosphorylation of glutamate receptors [32], and decreases in physiological excitability [33]. Sleep-wake alterations in synaptic strength are in turn paralleled by changes in broader aspects of cortical function, including a general dampening of activity propagation across the cortex during sleep relative to wakefulness [4]. Recent findings from our group support this proposition regarding systems-level changes in functional connectivity such that learning-related increased connectivity during wake is restored during subsequent sleep [34]. Although the implications of these changes for functional connectivity dynamics measured by fMRI have not been formally modeled, evidence that connectivity dynamics are “slower” during sleep compared to wakefulness seem consistent in spirit with known aforementioned aspects of sleep neurophysiology.

Interestingly, no connectivity states were specific either to sleep or wakefulness. In the context of a 7-state cluster solution, all participants expressed all states while asleep, and all but 2 participants expressed all states when awake. Although there were subtle differences in the frequency of specific states across sleep and wakefulness, there was no indication that sleep onset was accompanied by a fundamental change in the state repertoire. As such, the most pronounced differences between sleep and wakefulness were evident in temporal rather than spatial aspects of functional brain connectivity. Thus, a dynamic measure of sleep-wake states may be more appropriate for studying whole-brain connectivity.

Evidence that the spatial properties of the state repertoire were largely conserved across sleep and wakefulness contrasts with previous reports of marked changes in state topology given changes in awareness or arousal. In one report [35], dynamic connectivity states visited during anesthesia were topologically very similar to the underlying structural–white matter–skeleton, whereas connectivity states visited during awareness departed quite markedly from underlying structure. We found nothing analogous in the present study. Whether the differences in the two sets of findings relate to differences in species (i.e., monkey versus human), awareness manipulation (i.e., anesthesia versus sleep), analysis (i.e., comparison of time-resolved FC with structural connectivity matrix versus participant’s own mean), or something else, is not clear. Thus, the notion that dynamic functional connectivity states show more distant excursions (in a Euclidean sense) from the structural skeleton given changes in awareness and/or arousal remains compelling. However, predictions this notion makes about sleep/wake differences in human functional connectivity dynamics were not borne out in the present study.

Although differences between wake and sleep were most evident in dynamic aspects of functional connectivity, there were a selected number of static intra- and inter-network connections that varied across conditions. In both conditions, static FC was marked by strong positive correlations within functional networks, and weaker positive correlations and anti-correlations between functional families. Comparing conditions, there were a small number of inter-network connections that were stronger in NREM2 than Wake. These differences included stronger connections between three intra-visual networks. This result converges with previous findings that showed an increase in BOLD fluctuations within the visual network during light sleep when compared to wakefulness [36]. We also found stronger correlations in NREM2 between a cerebellar IC and a dorsal attention IC. Interestingly, previous findings reported an increase in FC within the dorsal attention network [37]; although our finding involved a cerebellar IC, evidence suggests that cerebellar nodes should be considered as part of the dorsal attention network [38]. Finally, we found a stronger correlation during NREM2 between a visual network IC and a subcortical IC, which has not been reported or examined before. This may be a neurophysiological biomarker of visual imagery during sleep; however, this association requires further research.

Dynamic FC revealed interesting temporal features of the data that would have otherwise been obscured using static methods. Our results suggest that: (a) dynamic fluctuations between inter-cortical and cortical-subcortical might be a useful marker of arousal; and (b) reduced connectivity between cognitive control and default mode networks might reflect reduced awareness. The latter is consistent with previous studies showing a decoupling of the anterior and posterior nodes of the DMN, observed only previously during deep sleep, which is restored again during REM sleep [39]. However, in the absence of sufficient lighter, NREM1 sleep or REM data (where awareness is greater than NREM2) in contrast to deep slow wave sleep (where conscious awareness is minimal), it is not possible to draw strong conclusions about the neural signatures of awareness from the current study. Future EEG-fMRI studies that are able to obtain such data would reveal valuable insights into the nature of brain connectivity states that reflect awareness of the environment and the self.

## Supporting information

S1 File Supplementary material regarding methodology and results of the study. (DOCX)

S2 File Figures relating to supplementary material and supporting figures for the main manuscript. (PDF)

## References

[1] Deco G, Hagmann P, Hudetz AG, Tononi G. Modeling Resting-State Functional Networks When the Cortex Falls Sleep: Local and Global Changes. Cereb Cortex. 2013 10.1093/cercor/bht176 23845770 PMC6317504

[2] Horovitz SG, Braun AR, Carr WS, Picchioni D, Balkin TJ, Fukunaga M, et al Decoupling of the brain’s default mode network during deep sleep. Proc Natl Acad Sci U S A. 2009;106: 11376–11381. 10.1073/pnas.0901435106 19549821 PMC2708777

[3] Tagliazucchi E, von Wegner F, Morzelewski A, Borisov S, Jahnke K, Laufs H. Automatic sleep staging using fMRI functional connectivity data. Neuroimage. 2012;63: 63–72. 10.1016/j.neuroimage.2012.06.036 22743197

[4] Massimini M;, Ferrarelli F;, Huber R;, Esser SK. Breakdown of Cortical Effective Connectivity During Sleep. Science (80-). 2005;309: 2228–2233. 10.1126/science.1117256 16195466

[5] Dang-Vu TT, Bonjean M, Schabus M, Boly M, Darsaud A, Desseilles M, et al Interplay between spontaneous and induced brain activity during human non-rapid eye movement sleep. Proc Natl Acad Sci U S A. 2011;108: 15438–15443. 10.1073/pnas.1112503108 21896732 PMC3174676

[6] Schabus M, Dang-Vu TT, Heib DPJ, Boly M, Desseilles M, Vandewalle G, et al The Fate of Incoming Stimuli during NREM Sleep is Determined by Spindles and the Phase of the Slow Oscillation. Front Neurol. 2012;3: 40 10.3389/fneur.2012.00040 22493589 PMC3319907

[7] Maquet P, Ruby P, Maudoux A, Albouy G, Sterpenich V, Dang-Vu T, et al Human cognition during REM sleep and the activity profile within frontal and parietal cortices: a reappraisal of functional neuroimaging data. Progress in brain research. 2005 pp. 219–595. 10.1016/S0079-6123(05)50016-5 16186026

[8] Tononi G, Cirelli C. Sleep and the price of plasticity: from synaptic and cellular homeostasis to memory consolidation and integration. Neuron. 2014;81: 12–34. 10.1016/j.neuron.2013.12.025 24411729 PMC3921176

[9] Diering GH, Nirujogi RS, Roth RH, Worley PF, Pandey A, Huganir RL. Homer1a drives homeostatic scaling-down of excitatory synapses during sleep. Science (80-). 2017;355: 511–515. 10.1126/science.aai8355 28154077 PMC5382711

[10] Fogel SM, Smith CT. The function of the sleep spindle: a physiological index of intelligence and a mechanism for sleep-dependent memory consolidation. Neurosci Biobehav Rev. 2011;35: 1154–1165. 10.1016/j.neubiorev.2010.12.003 21167865

[11] Peigneux P, Fogel S, Smith C. Chapter 22 –Memory Processing in Relation to Sleep. Principles and Practice of Sleep Medicine. Principles and Practice of Sleep Medicine. 2017 pp. 335–347.

[12] Rasch B, Born J. About Sleep’s Role in Memory. Physiol Rev. 2013;93: 681–766. 10.1152/physrev.00032.2012 23589831 PMC3768102

[13] Boly M, Perlbarg V, Marrelec G, Schabus M, Laureys S, Doyon J, et al Hierarchical clustering of brain activity during human nonrapid eye movement sleep. Proc Natl Acad Sci U S A. 2012;109: 5856–61. 10.1073/pnas.1111133109 22451917 PMC3326471

[14] Larson-Prior LJ, Zempel JM, Nolan TS, Prior FW, Snyder AZ, Raichle ME. Cortical network functional connectivity in the descent to sleep. Proc Natl Acad Sci. 2009;106: 4489–4494. 10.1073/pnas.0900924106 19255447 PMC2657465

[15] Tagliazucchi E, Crossley N, Bullmore ET, Laufs H. Deep sleep divides the cortex into opposite modes of anatomical???functional coupling. Brain Struct Funct. 2016;221: 4221–4234. 10.1007/s00429-015-1162-0 26650048

[16] Hutchison RM, Womelsdorf T, Gati JS, Everling S, Menon RS. Resting-state networks show dynamic functional connectivity in awake humans and anesthetized macaques. Hum Brain Mapp. 2013;34: 2154–2177. 10.1002/hbm.22058 22438275 PMC6870538

[17] Calhoun VD, Miller R, Pearlson G, Adali T. The Chronnectome: Time-Varying Connectivity Networks as the Next Frontier in fMRI Data Discovery. Neuron. 2014 pp. 262–274. 10.1016/j.neuron.2014.10.015 25374354 PMC4372723

[18] Allen EA, Damaraju E, Plis SM, Erhardt EB, Eichele T, Calhoun VD. Tracking Whole-Brain Connectivity Dynamics in the Resting State. Cereb Cortex. 2014; 24: 663–676. 10.1093/cercor/bhs352 23146964 PMC3920766

[19] Allen EA, Damaraju E, Eichele T, Wu L, Calhoun VD. EEG Signatures of Dynamic Functional Network Connectivity States. Brain Topography. 2017: 1–16. 28229308 10.1007/s10548-017-0546-2 PMC5568463

[20] Hutchison RM, Morton JB. Tracking the Brain’s Functional Coupling Dynamics over Development. J Neurosci. 2015;35: 6849–6859. 10.1523/JNEUROSCI.4638-14.2015 25926460 PMC6605187

[21] Abou-Elseoud A, Starck T, Remes J, Nikkinen J, Tervonen O, Kiviniemi V. The effect of model order selection in group PICA. Hum Brain Mapp. 2010;31: 1207–1216. 10.1002/hbm.20929 20063361 PMC6871136

[22] Xie H, Calhoun VD, Gonzalez-castillo J, Damaraju E, Miller R, Bandettini PA, et al NeuroImage Whole-brain connectivity dynamics reflect both task-specific and individual-specific modulation: A multitask study. 2018;180: 495–504. 10.1016/j.neuroimage.2017.05.050 28549798 PMC5700856

[23] Cash SS, Halgren E, Dehghani N, Rossetti AO, Thesen T, Wang CM, et al The human K-complex represents an isolated cortical down-state. Science (80-). 2009 10.1126/science.1169626 19461004 PMC3715654

[24] Csercsa R, Dombovári B, Fabó D, Wittner L, Erss L, Entz L, et al Laminar analysis of slow wave activity in humans. Brain. 2010 10.1093/brain/awq169 20656697 PMC3105490

[25] Massimini M. The Sleep Slow Oscillation as a Traveling Wave. J Neurosci. 2004 10.1523/JNEUROSCI.1318-04.2004 15295020 PMC6729597

[26] Mölle M, Marshall L, Gais S, Born J. Grouping of spindle activity during slow oscillations in human non-rapid eye movement sleep. J Neurosci. 2002. 10.1523/JNEUROSCI.22-24-10941.2002 PMC6758415 12486189

[27] Terzano MG, Mancia D, Salati MR, Costani G, Decembrino A, Parrino L. The cyclic alternating pattern as a physiologic component of normal NREM sleep. Sleep. 1985;8: 137–45. 10.1093/sleep/8.2.137 4012156

[28] Parrino L, Boselli M, Spaggiari MC, Smerieri A, Terzano MG. Cyclic alternating pattern (CAP) in normal sleep: Polysomnographic parameters in different age groups. Electroencephalogr Clin Neurophysiol. 1998;107: 439–450. 10.1016/s0013-4694(98)00108-4 9922091

[29] Terzano MG, Parrino L, Smerieri A, Chervin R, Chokroverty S, Guilleminault C, et al Erratum: “Atlas, rules, and recording techniques for the scoring of cyclic alternating pattern (CAP) in human sleep” (Sleep Medicine (2001) vol. 2 (6) (537–553)). Sleep Med. 2002;3: 185 10.1016/S1389-9457(02)00004-7 14592270

[30] McIntosh AR, Kovacevic N, Itier RJ. Increased brain signal variability accompanies lower behavioral variability in development. PLoS Comput Biol. 2008;4 10.1371/journal.pcbi.1000106 18604265 PMC2429973

[31] Saxe GN, Calderone D, Morales LJ. Brain entropy and human intelligence: A resting-state fMRI study. PLoS One. 2018;13 10.1371/journal.pone.0191582 29432427 PMC5809019

[32] Hinard V, Mikhail C, Pradervand S, Curie T, Houtkooper RH, Auwerx J, et al Key Electrophysiological, Molecular, and Metabolic Signatures of Sleep and Wakefulness Revealed in Primary Cortical Cultures. J Neurosci. 2012;32: 12506–12517. 10.1523/JNEUROSCI.2306-12.2012 22956841 PMC6621272

[33] Huber R, Mäki H, Rosanova M, Casarotto S, Canali P, Casali AG, et al Human cortical excitability increases with time awake. Cereb Cortex. 2013;23: 332–338. 10.1093/cercor/bhs014 22314045 PMC3539451

[34] Vahdat S, Fogel S, Benali H, Doyon J. Network-wide reorganization of procedural memory during NREM sleep revealed by fMRI. Elife. 2017;6 10.7554/eLife.24987 28892464 PMC5593513

[35] Barttfeld P, Uhrig L, Sitt JD, Sigman M, Jarraya B. Correction for Barttfeld et al., Signature of consciousness in the dynamics of resting-state brain activity. Proc Natl Acad Sci. 2015;112: E5219–E5220. 10.1073/pnas.1515029112 25561541 PMC4311826

[36] Horovitz SG, Fukunaga M, de Zwart JA, van Gelderen P, Fulton SC, Balkin TJ, et al Low frequency BOLD fluctuations during resting wakefulness and light sleep: A simultaneous EEG-fMRI study. Hum Brain Mapp. 2008;29: 671–682. 10.1002/hbm.20428 17598166 PMC6871022

[37] Larson-Prior LJ, Zempel JM, Nolan TS, Prior FW, Snyder AZ, Raichle ME. Cortical network functional connectivity in the descent to sleep. Proc Natl Acad Sci. 2009;106: 4489–4494. 10.1073/pnas.0900924106 19255447 PMC2657465

[38] Brissenden JA, Levin EJ, Osher DE, Halko MA, Somers DC. Functional Evidence for a Cerebellar Node of the Dorsal Attention Network. J Neurosci. 2016;36: 6083–6096. 10.1523/JNEUROSCI.0344-16.2016 27251628 PMC4887569

[39] Sämann PG, Wehrle R, Hoehn D, Spoormaker VI, Peters H, Tully C, et al Development of the brain’s default mode network from wakefulness to slow wave sleep. Cereb Cortex. 2011;21: 2082–2093. 10.1093/cercor/bhq295 21330468

