# Supplementary material for: Functional connectivity dynamics slow with descent from wakefulness to sleep
Source: PLoS One. 2019 Dec 2;14(12):e0224669. doi: 10.1371/journal.pone.0224669 (PMC6886758; doi:10.1371/journal.pone.0224669)
Supplement: S1 File — (DOCX) [file pone.0224669.s001.docx]

**Supplementary Methods and Materials**

**Participants**

Participants were required to meet the MRI safety screening criteria. Participants who passed the initial screening were then assessed using neuropsychological questionnaires. Participants had to score 10 or lower on the Beck Depression (Beck, Rial, & Rickels, 1974; Beck, Steer, & Garbin, 1988) and the Beck Anxiety (Beck, Epstein, Brown, Steer, & others, 1988); as well, participants must demonstrate no history or signs of sleep disorders as measured using the Sleep Disorders Questionnaire (4). Extreme morning and evening types were excluded according to the Morningness-Eveningness Questionnaire (Horne &Ostberg, 1976). All participants provided written informed consent and were financially compensated.

**Experimental Procedure**

Simultaneous EEG-fMRI data was recorded while participants were instructed to remain awake in the scanner for the wake session (8 min, 32 s) with their eyes closed, and then they were asked to sleep until awaken at the end of the scanning time.

**Data Acquisition and Preprocessing**

**EEG-fMRI Data Acquisition**

A 3.0T Magnetom Prisma magnetic resonance imaging system (Siemens, Erlangen, Germany) and a 64-channel radio frequency head coil were used to acquire imaging data. At the beginning of the scan, a structural T1-weighted image was acquired using a 3D MPRAGE sequence with TR = 2300 ms, TE = 2.98 ms, TI = 900 ms, FA = 9^o^, 176 slices, FoV = 256 mm x 256 mm, matrix size = 256 x 256, yielding a voxel size 1 mm x 1mm x 1mm. T2*- weighted functional images were acquired during wake and sleep sessions with a gradient echo-planar sequence using axial slice orientation with TR = 2160 ms, TE = 30 ms, FA = 90 ^o^, 40 transverse slices, 3 mm slice thickness, 10% inter-slice gap, FoV = 220 mm x 220 mm, matrix size = 64 x 64, yielding a voxel size = 3.44 mm x 2.44 mm x 3 mm. To synchronize the EEG and MRI recordings, a Sync Box was used. This hardware is required to reduce residual artefact from inaccurate sampling of the MRI gradient artefact. However, hardware synchronization does not completely resolve this problem as the template used to correct the gradient artefact can also drift due to small imprecisions in the TR that depend on the type of slice acquisitions used. Selecting MRI sequence parameters that are time-stable resolves this issue. The image acquisition artefact will be stationary if the actual TR matches a common multiple of the EEG sample time (0.2 ms for 5 kHz), the product of scanner clock precision (0.1 μs for 10 MHz),x and number of slices. Thus, the aforementioned sequence parameters were chosen to allow for simultaneous EEG recording with a time stabilized gradient artifact, and to ensure that the gradient artifact harmonic (18.52 Hz) would be as high as possible, and away from slower, sleep-related EEG frequencies of interest. A 2160 ms repetition time was chosen to match EEG sample time (common multiple of 0.2 ms), scanner clock precision product (0.1 μs), and the total number of slices – 40 – used (5).

A 64-channel magnetic resonance (MR) compatible EEG cap [including one ECG lead, Brain MR, Easycap, Herrsching, Germany] and two MR-compatible 32-channel amplifiers (Brainamp MR plus, Brain Products GmbH, Gliching, Germany) were used to acquire simultaneous EEG during the scanning session. Participants’ heads were restrained and cushioned using comfortable foam to minimize head movements in the scanner and to reduce motion-related EEG and fMRI artifacts. MRI sequence parameters were chosen to ensure that the lowest residual gradient artifacts (18.52 Hz) would not compromise sleep-related EEG of interest. In addition, we repositioned participants in the MRI scanner so that the subjects were shifted away from iso-centre of the magnetic field by 40mm, as recommended by Mullinger et al. 2011. At this position, the MRI images are not impacted, but the ballistocardiographic (BCG) artifact has been reported to be reduced by up to 40%, making subsequent artifact correction more effective. Scalp electrodes were referenced to FCz, and two bipolar ECG recordings from V2 – V5 and from V3 – V6 using an MR-compatible 16-channel bipolar amplifier (Brainamp ExG MR, Brain Products GmbH, Gliching, Germany). Importantly, these additional ECG recordings provided better visualization of the QRS complex such that r-peaks could be more easily and precisely detected for use in subsequent BCG artifact correction. Electrode-skin impedance was reduced to < 5 kOhm using high-chloride abrasive electrode paste (Abralyt 2000 HiCL; Easycap, Herrsching, Germany).

EEG was acquired at a 5000-samples per second rate and was digitized with a 500-nV resolution. An analog filter using a band-limiter low pass filter (500 Hz) and a high pass filter (10s corresponding to 0.0159Hz) was applied on EEG data. A fiber optic cable transferred EEG and ECG recordings to a personal computer that was synchronized to the scanner’s clock using a Brain Products Recorder Software, Version 1.0x (Brain Products, Gilching, Germany).

To remove EEG artifacts due to scanning, a three-step process was implemented. This first involved the removal of MRI gradient artifacts using an adaptive template subtraction method and down-sampling to 250 Hz (Allen, Josephs, & Turner, 2000). This step was performed in Brain Products Analyzer. The second step involved the removal of ballistocardiographic artifacts using template subtraction that are time-locked to the R-peak of the QRS complex of the cardiac rhythm. The second step was performed using semi-automatically detected and visually verified r-peaks in the ECG recording. Each and every r-peak was visually inspected and adjusted if necessary to ensure accurate r-peak detection, and to correct both false positives and false negative r-peak detections, in order to optimize subsequent BCG correction. After the initial BCG correction using average artifact subtraction (Allen, Polizzi, Krakow, Fish, & Lemieux, 1998), we verified the visual quality of data, inspected the amplitude of the residual artifacts time-locked to the r-peaks (SI Figure 4). Independent component analyses (ICA) (Mantini et al., 2007; Srivastava et al., 2005) was applied to remove remaining BCG residual artifact if the peak of the maximum amplitude of the residual artifact exceeded 3 μV during the QRS complex (e.g., 0 to 600ms). Finally, a low-pass filter (60 Hz) was applied to the EEG data, which were then re-referenced to averaged mastoids.

Following artifact correction (see SI Figure 5 for an example of EEG quality during sleep), standard sleep stage scoring was performed (11) using the VisEd Marks toolbox (https://github.com/jadesjardins/vised_marks) in EEGLAB (12). Thirty-second long EEG epochs were classified into the different sleep stages [i.e., non-rapid eye movement sleep (NREM) Stages wake, NREM 1, 2, 3 and REM]. Participants had an average of 419 volumes classified as NREM2. Only six participants had a sufficient number of volumes in NREM3 sleep; thus, to maintain sufficient statistical power, dynamics analyses compared only Wake *vs*. NREM2.

Functional images were preprocessed using SPM12 (http://www.fil.ion.ucl.ac.uk/spm/software/spm12/) in MATLAB (version 9.6.1 R2016b). Standard preprocessing procedures included realignment using rigid body transformations and reslicing; coregistration of the mean realigned image to the structural T1-image; spatial normalization of the resultant volumes into Montreal Neurological Institute (MNI152) space; and smoothing with a Gaussian kernel (FWHM = 8 mm).

Of the 35 participants, 21 were included in the analysis who had sufficient sleep volumes (i.e., > 5 minutes of consolidated sleep), and whose volumes were not contaminated by motion artifacts (translation cutoff = 1.5mm, rotation cutoff = 1.5 degrees). Two of the 21 participants had 150 wake volumes; the remaining 19 had 220 volumes. Each participant’s sleep stage onsets and durations were used to categorize functional volumes acquired during the sleep session. Sleep volumes were separated into Wake, NREM1, NREM2, NREM3, and REM sleep for subsequent analyses. Participants had an average of 419 volumes classified as NREM2. SI Table 1 summarizes the average time spent in each stage of sleep and wakefulness during the sleep session.

**Stationary Mean FC Estimation**

We estimated for each condition *c* a correlational matrix C*_c_*, to visualize and compare stationary connections in Wake and NREM2. Full time courses of all 42 ICs for each subject (*s*) were cross-correlated with one another to compute stationary mean FC in Wake and NREM2. Group stationary mean FC in Wake and Sleep were then computed by taking the average cross-correlations across subjects. Each matrix was vectorized and a spatial correlation was computed between the stationary FC in Wake and NREM2 vectors to assess their similarity. Furthermore, to better visualize stationary FC differences across conditions, a difference matrix was computed (C_NREM2_ – C_Wake_). To test for statistical significance of connectivity between ICs, *t* tests were performed with the null hypothesis of zero correlation on the difference matrix. To correct for multiple comparisons, the false discovery rate (FDR) method was used with a *P* value of .01.

**Dynamic FC Estimation**

To compute dynamic FC, a sliding window (width = 15 TRs, 1 TR time step) was used to obtain internetwork dynamic FC (Allen et al., 2014). All 42 ICs in each window were cross-correlated using Pearson’s correlation resulting in covariance matrices. Resultant window (W) matrices representing the total number of unique elements (N), such that each element (n) represents a covariance between two ICNs. All windows across subjects and condition were concatenated into a large matrix composed of rows equal to total number of windows and columns equal to N.

$$N=\frac{n(n-1)}{2}$$

After subject level demeaning, the concatenated matrix was then submitted into a k-means clustering algorithm to find recurrent patterns in FC (14) across all participants in wake and sleep. The squared Euclidean distance metric was used to evaluate distances between windowed covariance matrices. The clustering algorithm was computed using values of k ranging from 2 to 21 to ensure that the resultant functional connectivity states were consistent across varying k-values. Spatial correlations of qualitatively similar connectivity states across different k-solutions were computed to confirm that they are indeed similar. Furthermore, spatial correlations were computed between all other connectivity states to confirm that the states that were qualitatively identified as similar were not also similar to other states. A k-7 solution was chosen for further dynamics analyses (Allen et al., 2014; Barttfeld, Uhrig, Sitt, Sigman, & Jarraya, 2015). The reliability of the connectivity state frequency of expression metric (see SI Results below) was also computed on varying ranges of k-solutions to confirm that the dynamic metrics presented in this paper are not biased to a k-7 solution.

k-means clustering produces two outcomes of interest that were used to compute dynamic metrics that elucidated temporal features of the data. First, a C-matrix composed of k-rows and columns equal to the number of unique elements in the FC matrices was generated. Each row represented a connectivity state that was identified by k-means as a recurrent pattern in the data. Second, an IDX vector, or a window state label vector, was generated that classified each window as a connectivity state (see SI Figure 2 for an example). The IDX vectors of the longest consecutive chain or chains of windows present per participant were used to calculate dynamic metrics of interests to preserve temporal features of the data.

**Estimating Heart Rate**

Using the participants’ ECG data, heart rate was estimated by computing the R-R interval during Wake and NREM2. Because there were various durations and onsets of NREM2 across participants, average heart rate throughout all NREM2 was computed for each participant.

**Supplementary Results**

**Spatial Correlations**

To validate the robustness of the connectivity states that were reported using a k = 7 solution, we computed spatial correlations between connectivity states that qualitatively look similar from k solution of 2 to 21 (see SI Figure 3 for an example). Furthermore, this process was repeated iteratively across all other connectivity states to confirm their dissimilarity to connectivity states that are qualitatively dissimilar.

**Frequency of State Expression Difference**

We computed difference scores between the frequencies of expression of similar connectivity states that were reported. Here, we show the difference score of frequencies of connectivity states obtained from a k5 and k15 solution. We show that there are no significant differences in the difference scores for each of the connectivity state that was assessed (see SI Figure 6). Specifically, we show a non-significant effect of connectivity state, *F*(1) = 2.72, *ns*, and a non-significant effect of condition (Wake, NREM2), *F*(1) = .71, *ns*, for difference scores regarding connectivity state-1; and a non-significant effect of connectivity state, *F*(1) = 2.47, *ns*, and a non-significant effect of condition (Wake, NREM2), *F*(1) = .08, *ns*, for difference scores regarding connectivity state-5. Further, for connectivity state-6, difference scores were only computed between a k7 and k15 solution because this connectivity state was not present in a k5 solution. We also found a non-significant difference *t*(20) = .51, *ns,* between the difference scores of connectivity state-6 in a k7 and k15 clustering solution.

**Heart Rate Variability**

Participants’ heart rate in Wake and NREM2 did not significantly differ t(20) = 1.568, *ns* (SI Figure 5).

**References**

1. Beck a T, Rial WY, Rickels K. Short form of depression inventory: cross-validation. Vol. 34, Psychological reports. 1974. p. 1184–6.

2. Beck AT, Steer RA, Garbin MG. Psychometric properties of the Beck Depression Inventory: Twenty-five years of evaluation. Clin Psychol Rev [Internet]. 1988;8(1):77–100. Available from: citeulike-article-id:9156523%5Cnhttp://dx.doi.org/10.1016/0272-7358(88)90050-5

3. Beck AT, Epstein N, Brown G, Steer RA, others. An inventory for measuring clinical anxiety: Psychometric properties. J Consult Clin Psychol [Internet]. 1988;56(6):893–7. Available from: http://www.ncbi.nlm.nih.gov/sites/entrez?Db=pubmed&DbFrom=pubmed&Cmd=Link&LinkName=pubmed_pubmed&LinkReadableName=Related Articles&IdsFromResult=3204199&ordinalpos=3&itool=EntrezSystem2.PEntrez.Pubmed.Pubmed_ResultsPanel.Pubmed_RVDocSum

4. Douglass AB, Bornstein R, Nino-Murcia G, Keenan S, Miles L, Zarcone VP, et al. The Sleep Disorders Questionnaire. I: Creation and multivariate structure of SDQ. Sleep [Internet]. 1994;17(2):160–7. Available from: http://www.ncbi.nlm.nih.gov/pubmed/8036370

5. Multert C, Lemieux L. EEG-fMRI; Physiological Basis, Technique, and Applications. Book. 2009;538.

6. Mullinger KJ, Yan WX, Bowtell R. Reducing the gradient artefact in simultaneous EEG-fMRI by adjusting the subject’s axial position. Neuroimage. 2011;54(3):1942–50.

7. Allen PJ, Josephs O, Turner R. A Method for Removing Imaging Artifact from Continuous EEG Recorded during Functional MRI. Neuroimage [Internet]. 2000;12(2):230–9. Available from: http://linkinghub.elsevier.com/retrieve/pii/S1053811900905998

8. Allen PJ, Polizzi G, Krakow K, Fish DR, Lemieux L. Identification of EEG events in the MR scanner: the problem of pulse artifact and a method for its subtraction. Neuroimage. 1998;8(3):229–39.

9. Mantini D, Perrucci MG, Cugini S, Ferretti A, Romani GL, Del Gratta C. Complete artifact removal for EEG recorded during continuous fMRI using independent component analysis. Neuroimage. 2007;34(2):598–607.

10. Srivastava G, Crottaz-Herbette S, Lau KM, Glover GH, Menon V. ICA-based procedures for removing ballistocardiogram artifacts from EEG data acquired in the MRI scanner. Neuroimage. 2005;24(1):50–60.

11. Silber MH, Ancoli-Israel S, Bonnet MH, Chokroverty S, Grigg-Damberger MM, Hirshkowitz M, et al. The visual scoring of sleep in adults. Vol. 3, Journal of Clinical Sleep Medicine. 2007. p. 121–31.

12. Delorme A, Makeig S. EEGLAB: An open source toolbox for analysis of single-trial EEG dynamics including independent component analysis. J Neurosci Methods. 2004;134(1):9–21.

13. Allen EA, Damaraju E, Plis SM, Erhardt EB, Eichele T, Calhoun VD. Tracking Whole-Brain Connectivity Dynamics in the Resting State. Cereb Cortex [Internet]. 2014;24(3):663–76. Available from: http://www.cercor.oxfordjournals.org/cgi/doi/10.1093/cercor/bhs352

14. Lloyd SP. Least Squares Quantization in PCM. IEEE Trans Inf Theory. 1982;28(2):129–37.

15. Barttfeld P, Uhrig L, Sitt JD, Sigman M, Jarraya B. Correction for Barttfeld et al., Signature of consciousness in the dynamics of resting-state brain activity. Proc Natl Acad Sci [Internet]. 2015;112(37):E5219–20. Available from: http://www.pnas.org/lookup/doi/10.1073/pnas.1515029112
