# Supplementary material for: Functional connectivity dynamics slow with descent from wakefulness to sleep
Source: PLoS One. 2019 Dec 2;14(12):e0224669. doi: 10.1371/journal.pone.0224669 (PMC6886758; doi:10.1371/journal.pone.0224669)
Supplement: S2 File — (PDF) [file pone.0224669.s002.pdf]

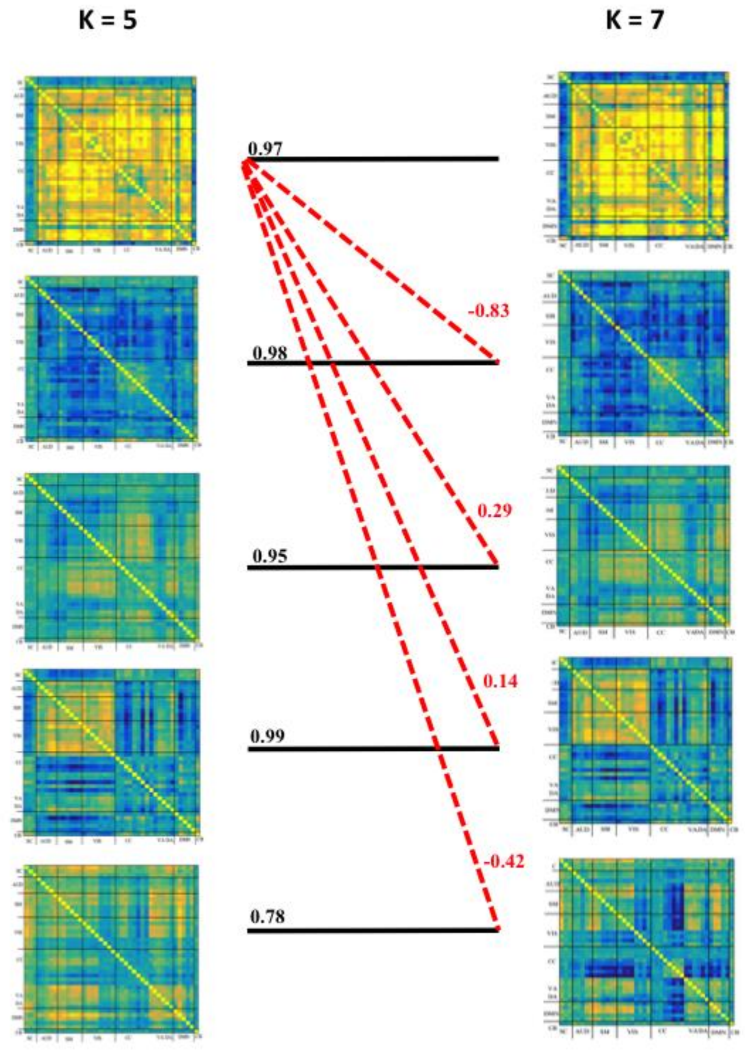

Figure 1. Spatial correlations comparing connectivity states from a k-5 solution and k-7 solution. Black lines connect qualitatively similar connectivity states. Red dashed lines show the spatial correlations of connectivity state-1 in a k-5 solution with connectivity states in a k-7 solution. Weaker correlations are indicative of the robustness of the connectivity states in varying k-solutions and the uniqueness of their spatial topologies.   Spatial correlations were iteratively computed between all connectivity states to confirm the similarity of selected connectivity states across varying k-solutions.

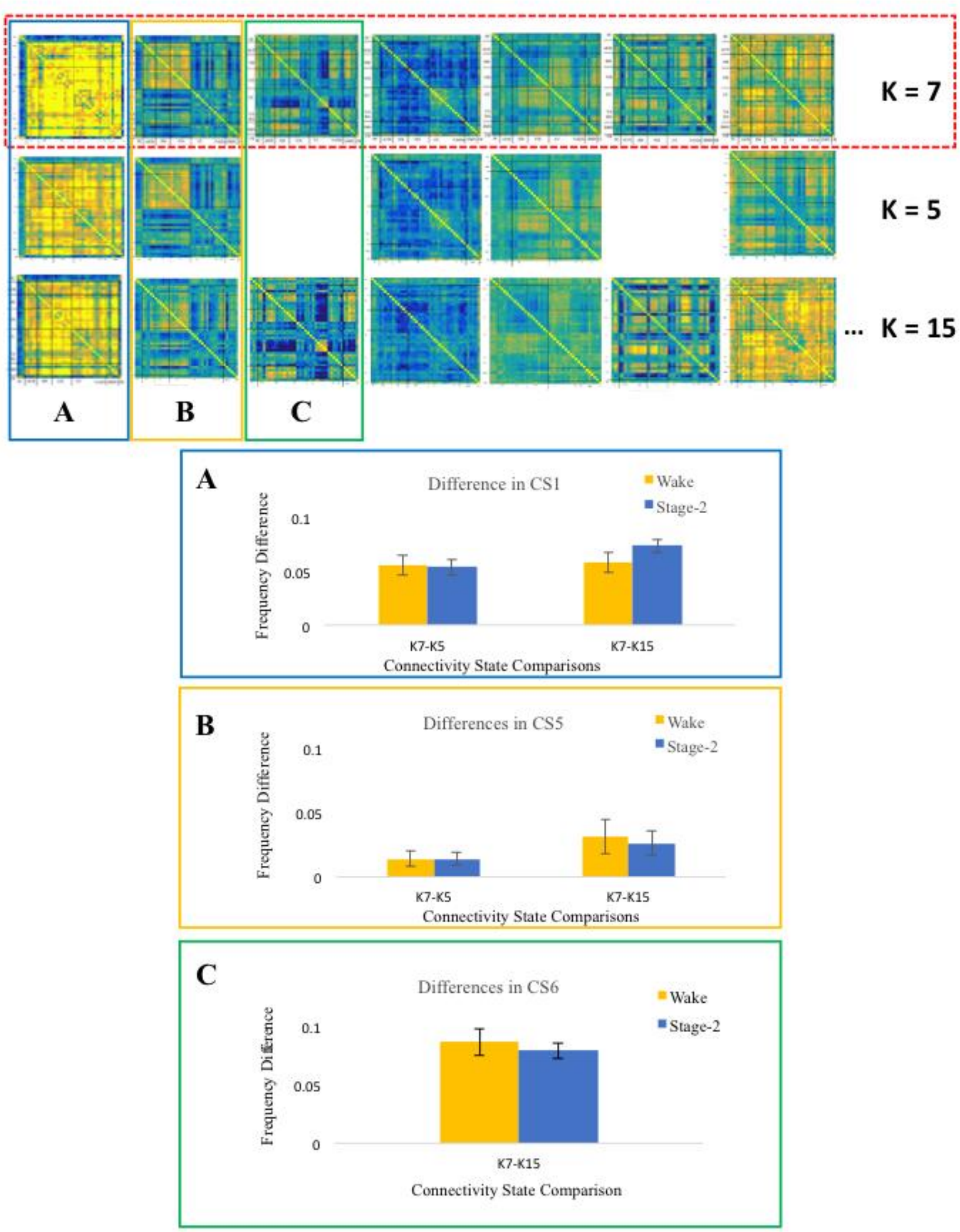

Figure 2. Difference scores were computed for connectivity states that showed statistically significant differences in Wake and NREM2. Top three panels show the connectivity states matrices derived from k-5, k-7, and k-15 solutions. The connectivity states were organized by grouping, column-wise, qualitatively similar states. Each group is highlighted and labeled as A, B, or C. Corresponding bar graphs are also labeled and highlighted with the same colour. The frequency of expression for connectivity state-1 (A), connectivity state-5 (B), and connectivity state-6 (C) in a k5 and k15 solution was subtracted from a k7 solution frequency score. Yellow

bars represent the difference scores during Wake, and blue bars represent the difference scores in NREM2. Frequency of expression did not change across k-solutions. This is indicative of the robustness of the dynamics analysis across different k solutions.

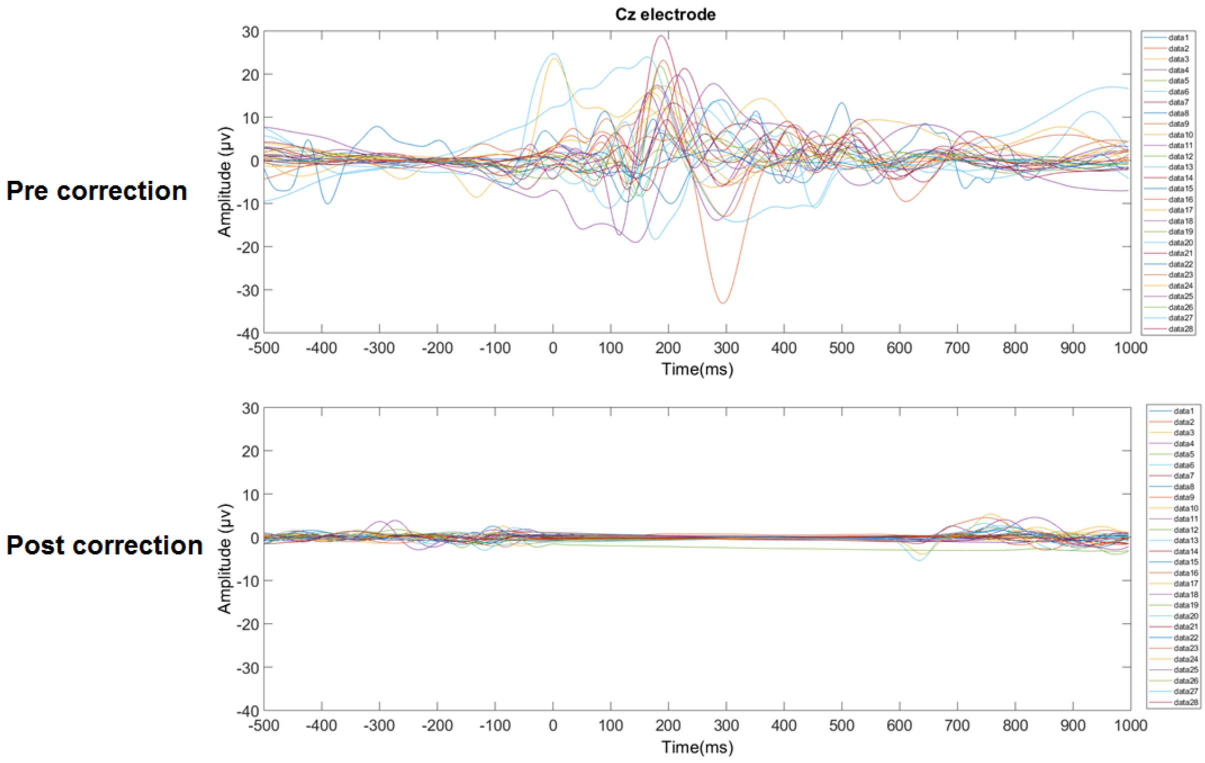

Figure 3. EEG data pre- and post- BCG correction.

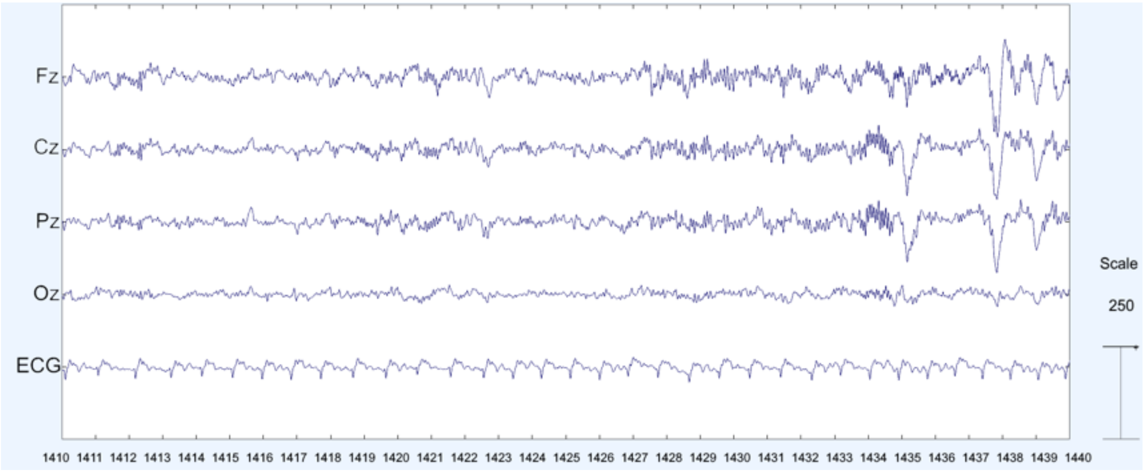

Figure 4. An example of the quality of the EEG and ECG data (from midline electrodes at Fz, Cz, Pz, and Oz) obtained during sleep in the MRI scanner for a 30 second epoch scored as NREM2 sleep following artifact correction, re-referencing and filtering.

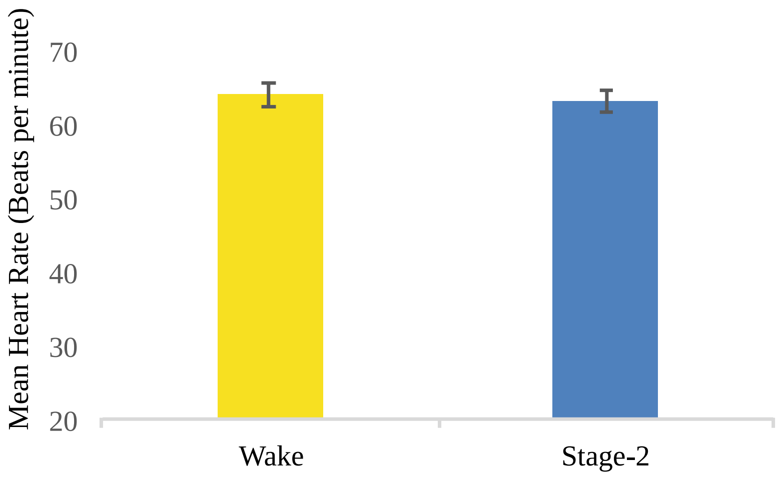

Figure 5. Mean heart rate (beats per minute) in Wake and NREM2. There was no difference in heart rate between conditions.

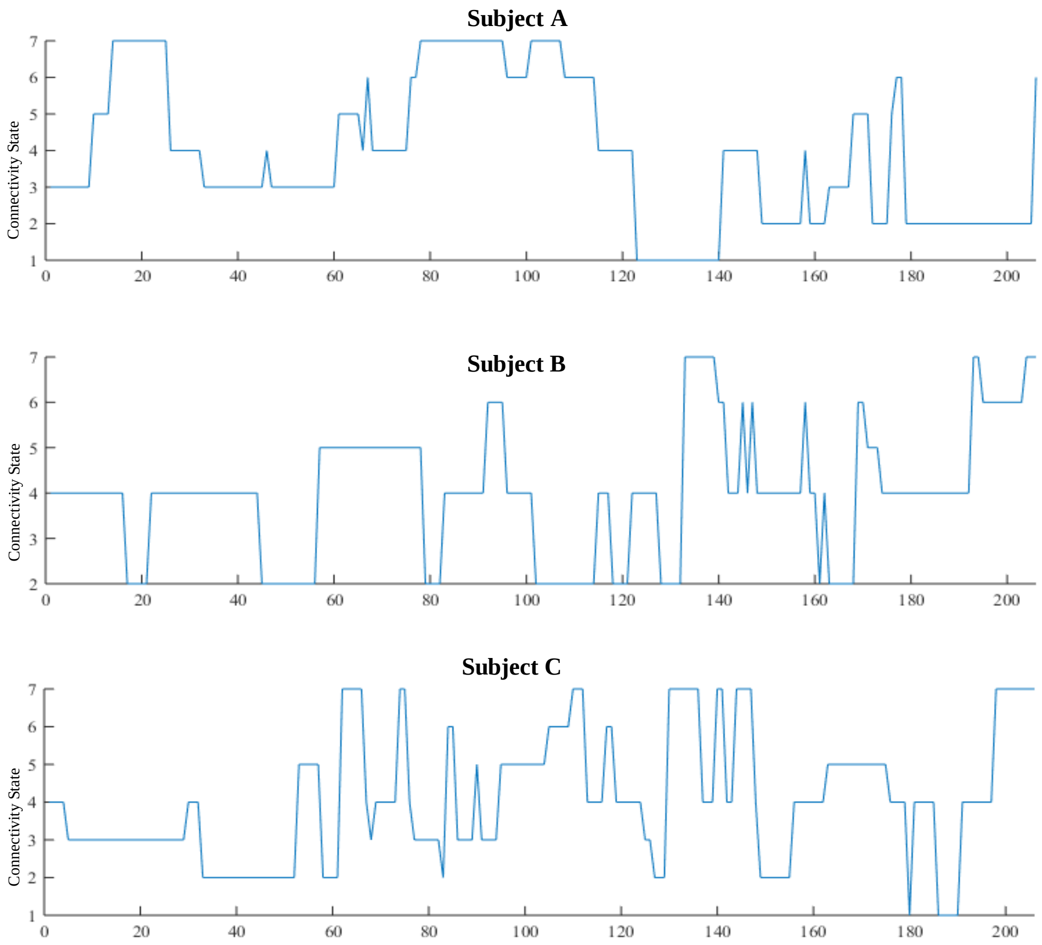

Figure 6. IDX vector of three representative participants during wakefulness. The number of windows is on the x-axis and the connectivity state is on the y-axis. Plots illustrate transitions between connectivity states. The IDX vector was used to derive dynamic metrics.

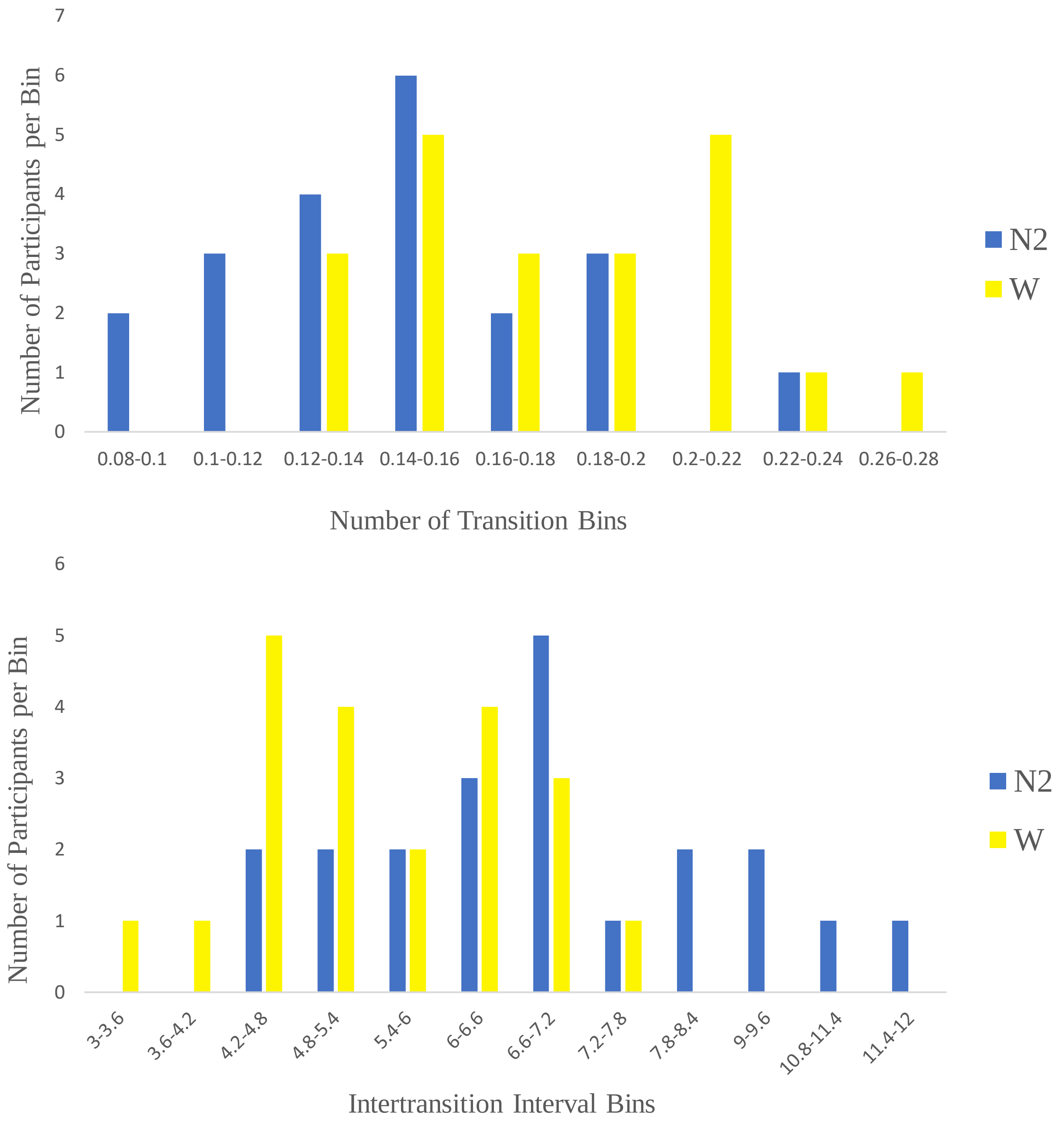

Figure 7. Histograms showing a near-gaussian distribution for the number of transition and intertransition interval dynamic metrics.

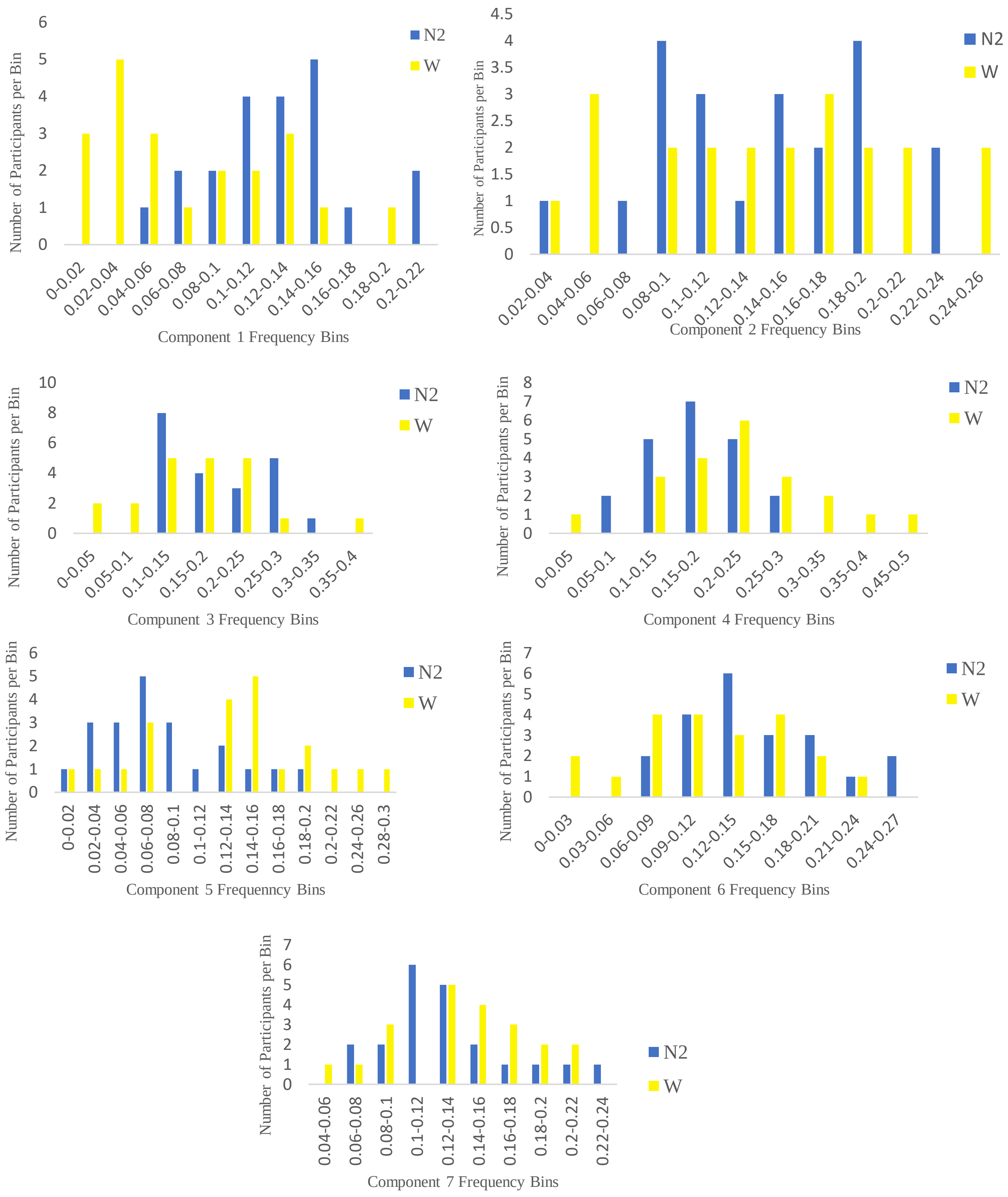

Figure 8. Histograms showing a near-gaussian distribution for the frequency of state expression dynamic metric.

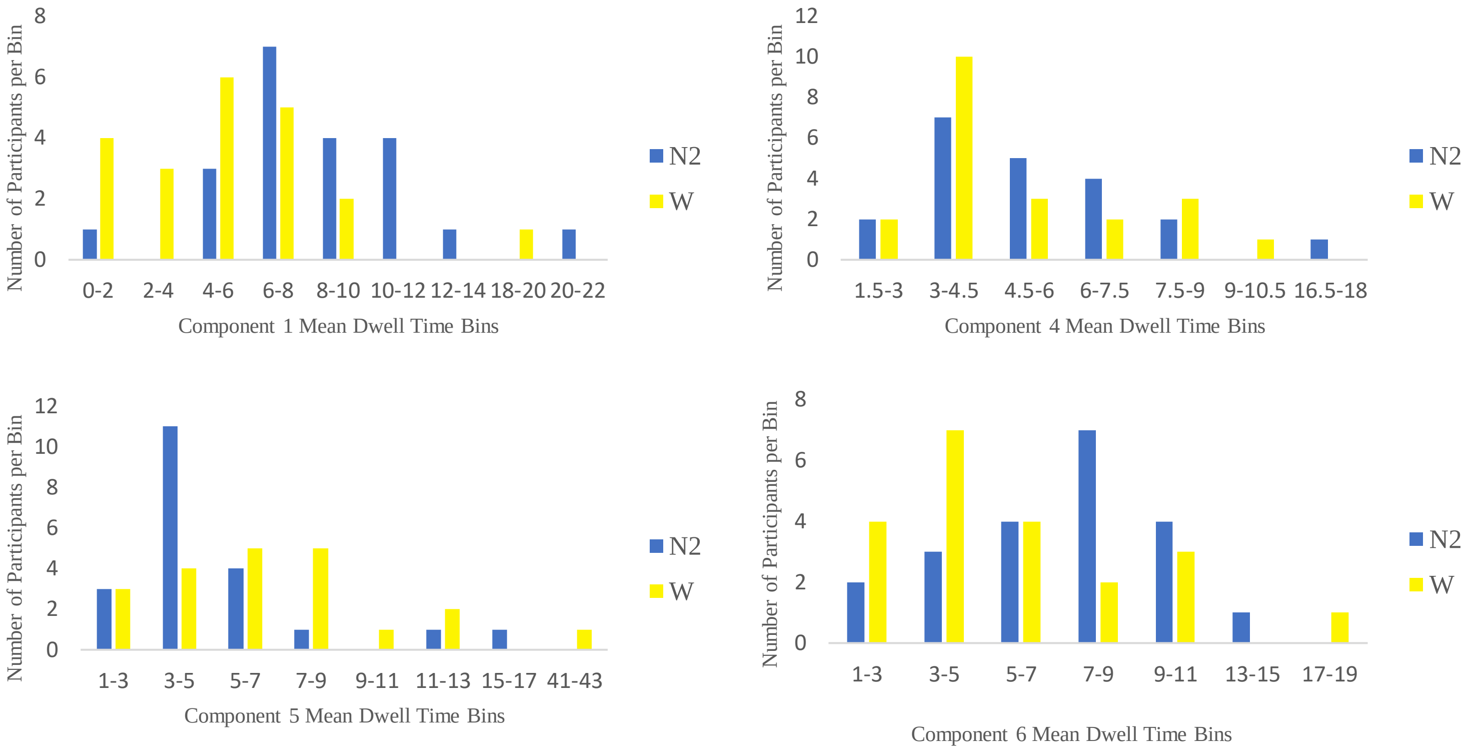

Figure 9. Histograms showing a near-gaussian distribution for the mean dwell time dynamic metric.

Table 1. Mean sleep architecture across participants

	**Mean (minutes)**	**Standard Deviation**	**Min**	**Max**
**Wake (n = 18)**	8.41	8.85	0.53	31.44
**NREM1 (n = 19)**	4.19	4.52	0.67	17.67
**NREM2 (n = 21)**	23.15	12.52	7.33	46.14
**NREM3 (n = 15)**	8.28	9.96	0.33	36.12
**REM (n = 2)**	7.33	0.46	7.00	7.67

Note: The average time and standard deviation in a given sleep stage across all participants included in the analyses that met our data inclusion criteria. Min values are reported for non-zero values.
